# Capacity of Lung Stroma to Educate Dendritic Cells Inhibiting Mycobacteria-Specific T-Cell Response Depends upon Genetic Susceptibility to Tuberculosis

**DOI:** 10.1371/journal.pone.0072773

**Published:** 2013-08-15

**Authors:** Marina A. Kapina, Elvira I. Rubakova, Konstantin B. Majorov, Nadezhda N. Logunova, Alexander S. Apt

**Affiliations:** Laboratory for Immunogenetics, Central Institute for Tuberculosis, Moscow, Russia; INRS - Institut Armand Frappier, Canada

## Abstract

The balance between activation and inhibition of local immune responses in affected tissues during prolonged chronic infections is important for host protection. There is ample evidence that regulatory, tolerogenic dendritic cells (DC) are developed and present in tissues and inhibit overwhelming inflammatory reactions. Also, it was firmly established that stromal microenvironment of many organs is able to induce development of immature regulatory DC (DCreg), an essential element of a general immune regulatory network. However, direct experimental data demonstrating inhibition of immune responses by stroma-instructed immature DCreg in infectious models are scarce, and virtually nothing is known about functioning of this axis of immunity during tuberculosis (TB) infection. In this study, we demonstrate that lung stromal cells are capable of supporting the development in culture of immature CD11b^+^CD11c^low^CD103^-^ DCreg from lineage-negative (lin^-^) bone marrow precursors. DCreg developed on lung stroma isolated from mice of genetically TB-hyper-susceptible I/St and relatively resistant B6 inbred strains inhibited proliferative response of mycobacteria-specific CD4^+^ T-cell lines a dose-dependent manner. Importantly, the inhibitory activity of B6 DCreg was substantially higher than that of I/St Dcreg. Moreover, when the donors of stromal cells were chronically infected with virulent mycobacteria, the capacity to instruct inhibitory DCreg was retained in B6, but further diminished in I/St stromal cells. DCreg-provided suppression was mediated by a few soluble mediators, including PGE_2_, NO and IL-10. The content of CD4^+^Foxp3^+^ Treg cells in the mediastinal, lung-draining lymph nodes at the advanced stages of chronic infection did not change in I/St, but increased 2-fold in B6 mice, and lung pathology was much more pronounced in the former mice. Taken together, these data provide genetic evidence that the capacity to maintain populations of regulatory cells during *M. tuberculosis* infection is a part of the host protective strategy.

## Introduction

Dendritic cells (DC) are the most potent initiators of adaptive immune responses, but also are able to establish and maintain immunological non-responsiveness or tolerance, especially at immature stages of differentiation when their patrol functions substantially excel T-cell-stimulatory ones [1]. This tolerogenic capacity of immature regulatory DC (DCreg) is released through a plethora of mechanisms, including the production of inhibitory cytokines, induction of anergy and instruction of regulatory T cells [2,3]. Such a multilayered system of immune response inhibition apparently underlies indispensable necessity to prevent not only detrimental immunity to self, gut-flora and non-pathogenic respiratory antigens, but also excessive inflammatory, tissue damaging responses to invading pathogens [4]. Of particular importance is the balance between activation and inhibition of immune responses during prolonged chronic infections when the tissue-damage/loss-of-function payment for a diminished multiplication and dissemination of the parasite may appear inadmissible.

Pulmonary tuberculosis (TB) is an outstanding example of such infections. It is generally accepted that following successful establishment of infection caused by *M. tuberculosis* a complex pattern of cellular immune responses interlinking numerous cell subsets and soluble mediators is developed to activate bactericidal capacity of infected macrophages and to contain the spread of mycobacteria to yet non-affected zones of the lung and extra-pulmonary locations [5-7]. However, this infection-restricting function of strong cellular responses in many cases is only temporary beneficial for the host. Phagocytes and lymphocytes entering the lung tissue rapidly form granulomata. In genetically TB-susceptible hosts granulomatous response is not containment and is often combined with and/or replaced by diffuse, sometimes caseous, TB pneumonia severely affecting the breathing function of the lung. Both in humans and mice, granulomas progressively grow and develop necrotic centers surrounded by inflamed and hypoxic tissue [8-10]. Thus, maintenance of the balance between protection and pathology – both mediated by cellular immune responses – is essential to successfully resist the disease triggered by *M. tuberculosis* [11]. Ability of regulatory cells of immune system, including DCreg, to inhibit overwhelming cellular responses is considered as an important strategic element of defense. Mycobacterial infections are excellent models to study the balance between protective immunity and immune-mediated pathology because it needs to be maintained for prolonged periods of time.

There is ample evidence that stromal microenvironment of many organs is able to induce DCreg development [12-15], and it is thought that within tissues DCreg locally terminate immune responses to return homeostasis following pathogen invasion [16]. However, direct experimental data demonstrating inhibition of immune responses by immature CD11b^+^ DC in infectious models are scarce. In a seminal work of Svensson et al. [17] it has been demonstrated for the first time that splenic stroma educated DCreg which produced IL-10 inhibiting T cell response, and that *L. donovani* infection increased the instructive capacity of stromal cells. More recently, it was shown that selective *in vivo* depletion of Langerhans DC, but not skin-derived DC of other types, augments the type 1 immune response during *L. major* infection and attenuates the disease in mice [18]; however, neither CD phenotype of these regulatory DC, nor possible role of their education by stromal cells were addressed in this work.

Although capacity of a cell line mimicking lung stroma to instruct development of DCreg with anti-inflammatory activity was clearly demonstrated [15], it remains unknown whether or not the *bona fide* pulmonary stromal cells educate inhibitory DCreg and do the latter play any role in regulating T cell responses against TB infection. More generally, data are lacking on possible role of DCreg during chronic bacterial infections, and the importance to gain relevant knowledge was recently emphasized [19]. Potential influence of the host genetic susceptibility to infection on parameters of DCreg induction/function was addressed to for the first time in *Leishmania* model [16]. Recently, the influence of genetic susceptibility to *M. tuberculosis* infection on the development of regulatory DC and Treg populations was analyzed in the mouse model [20], but in this study the authors characterized the population of recently described CD11b^-^CD11c^+^CD103^+^ αE-DC [21] but not classical immature CD11b^-^DC11c^+^ DCreg.

In this study, we demonstrate that lung stromal cells, representing a mixture of different cell types, including resident CD68^+^ macrophages, CD31^+^ endothelial cells and ER-TR7^+^ fibroblasts, were capable of supporting the development of DCreg from lineage-negative (lin^-^) bone marrow precursors. DCreg developed on lung stroma isolated from mice of I/St and B6 inbred strains (genetically TB-hyper-susceptible and relatively resistant, respectively) inhibited the response of isogenic CD4^+^ T-cell lines specific to mycobacterial antigens in a dose-dependent manner. Importantly, the inhibitory activity of B6 DCreg was substantially higher than that of their I/St counterparts. Moreover, when the donors of stromal cells were chronically infected with virulent mycobacteria, the capacity to instruct inhibitory DCreg was retained by B6, but further diminished in I/St stromal cells. Compared to pre-infected controls, the content of CD4^+^Foxp3^+^ Treg cells in the mediastinal, lung-draining lymph nodes at the advanced stages of chronic infection did not change in I/St, but increased 2-fold in B6 mice, and lung pathology was much more pronounced in the former mice. Taken together, these data provide genetic evidence that the capacity to maintain populations of regulatory cells during *M. tuberculosis* infection is a part of the host protective strategy, apparently released through inhibition of overwhelming inflammatory reactions.

## Results

### Phenotypic characteristics of lung stroma and DC developed on stromal cells

Whilst the diversity of dendritic cells and macrophages populating the mouse lung tissue has been characterized in considerable detail [22,23], cellular composition of murine lung stroma is less well defined. Thus, we first evaluated the content of key stromal cells in the lungs of B6 and I/St mice isolated by collagenase digestion and plastic adherence (Figure S1). These cells were heterogeneous and contained major populations of resident CD68^+^ macrophages (>50%) and CD31^+^ endothelial cells (~15%). Unlike spleen stromal cells comprising a lot of fibroblasts [17], only ~7% of ER-TR7^+^ fibroblasts were present among adherent lung cells. Similar phenotypes were expressed by stromal cells obtained from mice chronically infected with TB, with an enlargement of the CD68^+^ macrophage population up to 70% (data no shown). Importantly, no major differences in the stromal composition between infected and non-infected mice of the two strains were found, excluding at least one possible cause of interstrain differences in DCreg activity described below.

**Figure 1:**
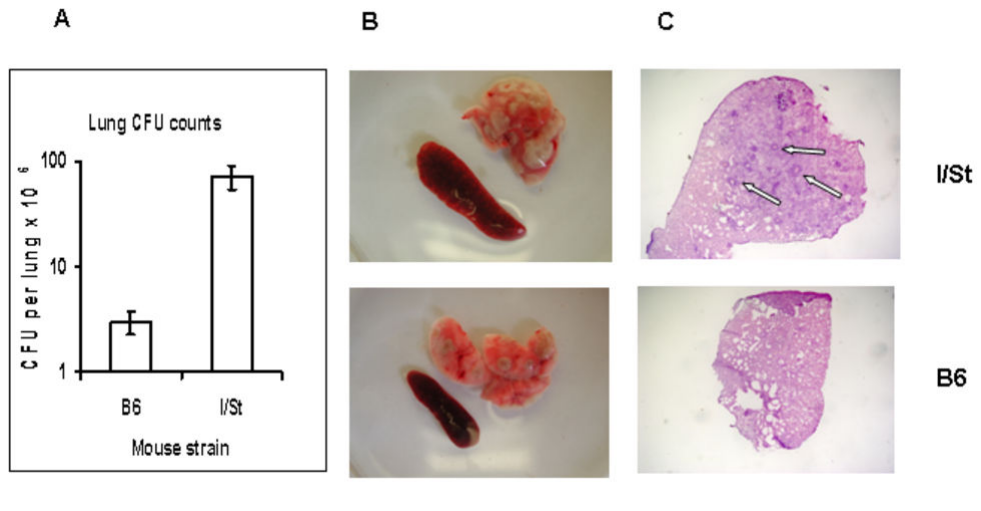
3 mo post aerosol infection with ~100 *M. tuberculosis* H37Rv CFU I/St mice display substantially more severe infectious course compared to B6 mice. (A) -1 log difference in lung CFU counts (N=5, P<0.001, ANOVA, 1 experiment of 3 similar); (B) – more prominent gross pathology of the lung and greater splenomegaly; (C) – granulomata with necrotizing centers (arrows) are present in the lungs of I/St but not of B6 mice (X25).

To find out whether lung stromal cells were able to support the in vitro differentiation of DC from the bone marrow precursors, we isolated progenitor cells from B6 and I/St mice using lineage depletion kit and co-cultured these cells (BM lin^-^ cells, ~60% c-kit^+^; Figure S2) with stromal cells for 7 days. Stromal cells were obtained from either naïve or TB-infected donors in order to determine possible influence of infection on the instructive capacity of stromal cells, especially since B6 and I/St mice substantially differ in severity of *M. tuberculosis*-triggered disease ( [24] and Figure 1). As shown in Table 1, by day 7 of co-culture, the majority of non-adherent cells expressed the CD11b^+^ phenotype, and were CD103^-^CD11c^-^MHCII^low/neg.^ , i.e., displayed the phenotype of immature DCreg [3]. The proportion of cells expressing co-stimulatory CD80 molecule (~20%) did not differ between mouse strains. The only statistically significant difference between groups was an enlargement of Gr1-positive cells (*P* < 0.05, ANOVA) in DCreg population after co-culturing with stroma from infected compared to naïve mice of both strains (Table 1). However, no interstrain differences regarding Gr-1^+^ cell content were noticed.

**Table 1:** Surface phenotype of non-adherent DCreg cells co-cultured with lung stroma for 7 days.*

DC surface marker	The source of “educating” lung stromal cells			
	I/St naive	I/St infected	B6 naive	B6 infected
CD11b	95.6 ± 3.6	91.6 ± 2.9	91.6 ± 3.9	95.3 ± 4.0
CD11c	9.8 ± 1.1	6.3 ± 1.6	7.8 ± 2.0	5.0 ± 1.9
Gr1	45.1 ± 5.0	58.7 ± 3.7	43.5 ± 4.4	54.4 ± 3.9
MHC Class II^high^	< 5	< 5	< 5	< 5
CD80	20.2 ± 2.1	18.7 ± 1.8	21.0 ± 1.7	22.1 ± 2.5
CD103	< 5	< 5	< 5	< 5

* Data are presented as the per cent of cells ± SD. For each experiment a mixture of bone marrow cells from 15–17 mice of each group (~3 x 10^8^) served as a source of Lin^-^ DC precursors (~2% = 4 x 10^6^). Phenotypes were assessed in 3-4 independent experiments and the results summarized. No statistically significant differences between groups were observed, except that Gr1-positive cells comprised significantly (*P* < 0.05, ANOVA) larger population when DC precursors were co-cultured with stroma from infected compared to naïve mice of both strains (see text).

### DCreg developed on stromal cells inhibit T cell response

**Figure 2:**
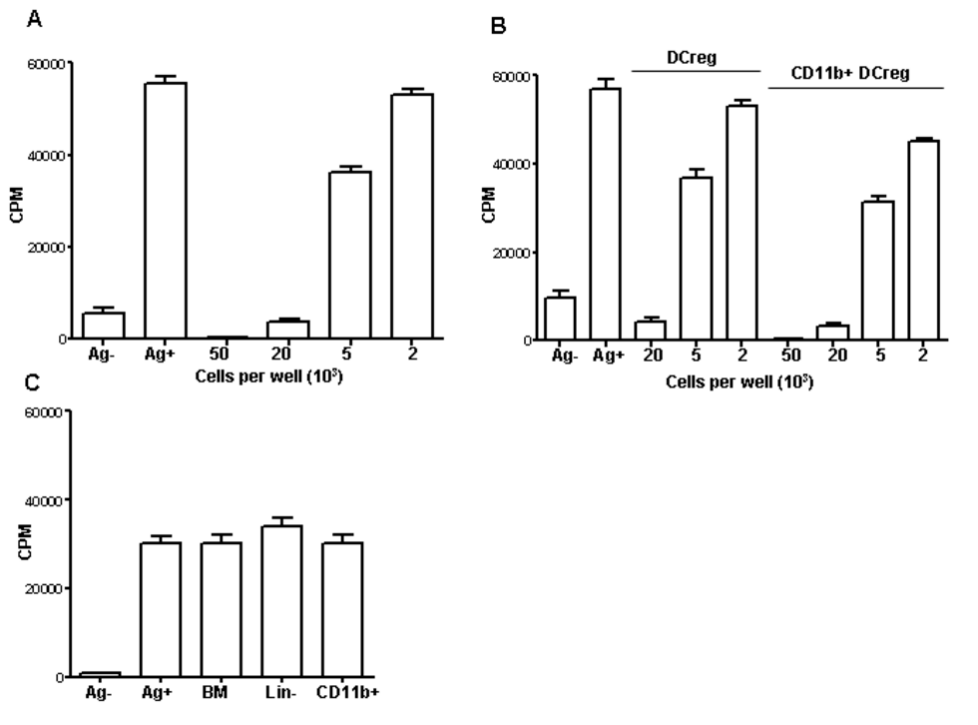
DCreg cells educated on lung stroma inhibit proliferative response of T cells specific to mycobacterial antigens. (A) - Addition of DCreg to co-cultures of T cell lines with APC suppresses antigen-specific proliferation in the dose-dependent manner (mean triplicate CFU counts ± SD from 4 independent experiments, ~90 per cent inhibition of proliferation by 20 x 10^3^ cells, ~40 per cent inhibition by 5 x 10^3^ cells, *P* < 0.001 and *P* < 0.01, respectively, compared to positive control). (B) - Suppressive capacity does not depend upon the admixture of other cell types since CD11b^+^ DCreg isolated by magnetic sorting (>95% purity) retain full inhibitory properties (mean triplicate CFU counts ± SD from 2 independent experiments, *P* < 0.001 and *P* < 0.01 for two doses of DCreg, ANOVA). (C) - Co-culture with lung stroma is requisite for acquisition of suppressive activity: total BMC, sorted CD11b^b^ BMC and freshly isolated lin^-^ cells did not inhibit T cell proliferation (mean triplicate CFU counts ± SD from 2 independent experiments, *P* > 0.3).

To determine if DC educated by stromal cells acquired regulatory activity, we tested their capacity to suppress proliferative response of T cell lines specific to a mixture of mycobacterial antigens. Non-adherent DC taken from co-cultures with lung stromal cells on day 7 of incubation effectively inhibited T cell proliferation in the dose-dependent manner (Figure 2A). To convince ourselves that inhibitory effect was not due to the admixture of stromal cells, we additionally purified the cultured CD11b^+^ population by magnetic sorting. When isolated cells (~96% CD11b^+^ purity) were added to T cell proliferation system their inhibitory capacity was similar to that of non-purified DC (Figure 2B), indicating that DCreg, but not occasionally captured stromal cells, were responsible for suppression.

The results described above were obtained using B6 mice, so we next tested whether stromal cells from I/St mice are also capable to instruct DCreg. In parallel, we tested if the bone marrow DCreg precursors possessed any suppressor activity regarding T cell proliferative response. As shown in Figure 2C, neither the total population of freshly isolated bone marrow cells, nor its purified lin^-^ and CD11b^+^ fractions possessed any inhibitory activity. On the other hand, I/St DCreg developed on stromal cells were suppressive (see below).

### TB infection diminishes the instructive capacity of I/St but not of B6 stromal cells

**Figure 3:**
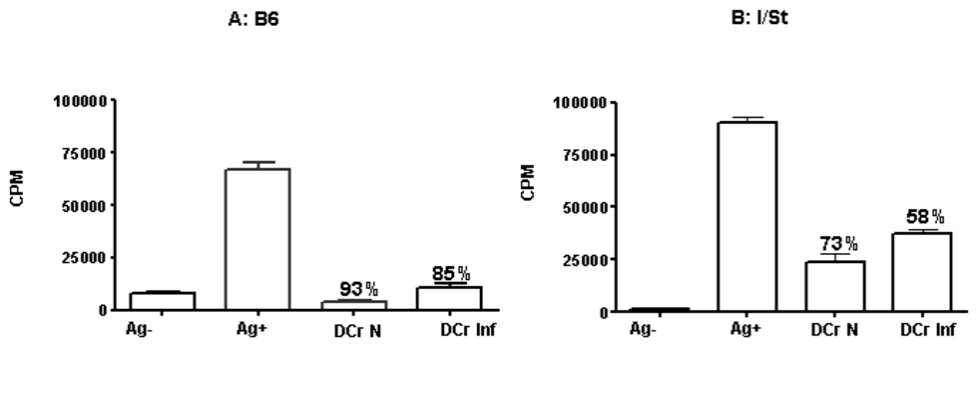
DCreg developed on lung stroma from naïve (N) TB-resistant (A) B6 mice are more potent inhibitors of T cell response than their counterparts from TB-susceptible (B) I/St mice (*P*<0.01, ANOVA). Inhibitory activity of DCreg developed on stromal cells from infected (Inf) animals was almost fully retained in B6 (A), but significantly (*P*<0.05, ANOVA) dropped in I/St mice (B). Mean triplicate CFU counts ± SD from 4 independent experiments and per cent of inhibition are displayed.

Pulmonary macrophages are the principle cell population which both fights against TB infection and grants a niche for mycobacterial intracellular survival and multiplication [25]. As shown above, these cells constitute the major population of lung stroma. Since B6 and I/St mice differ regarding their susceptibility to TB and degree of inflammation caused by infection (Figure 1), we evaluated whether lung stromal cells obtained from mice infected 3 mo earlier might differently be efficient at instructing progenitor cells along the DCreg pathway. Capacity of B6 DCreg to inhibit T cell proliferation was very strong and similar after education on lung stroma from naïve and TB-infected mice (Figure 3A). DCreg developed on lung stroma from naïve I/St mice were less effective inhibitors of T cell proliferation than their B6 counterparts (*P*<0.01, ANOVA). Furthermore, when I/St DCreg were developed on stromal cells from infected animals their inhibitory activity appeared to be significantly (*P*<0.05, ANOVA) weaker (Figure 3B). Thus, in genetically TB-resistant animals chronic infection did not alter the ability of lung stroma to regulate cellular immunity for a long period of time, whereas during a more severe infectious process stromal cells from affected organ partly lost their regulatory capacity.

### Inhibitors of T cell response

**Figure 4:**
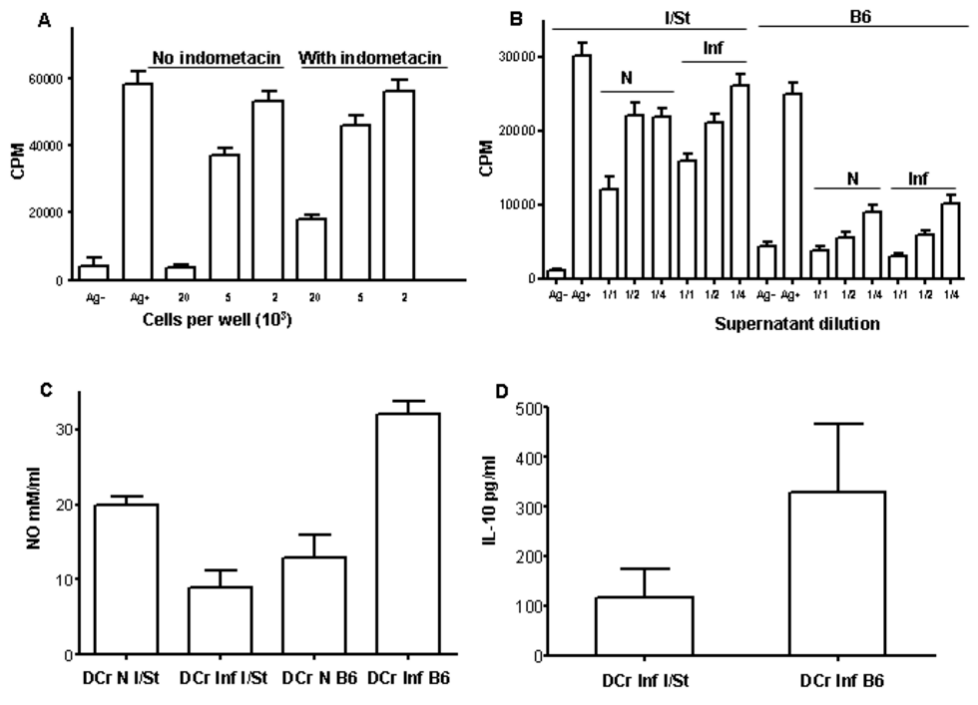
Soluble mediators of DCreg suppressor activity. (A) - B6 DCreg with blocked PGE_2_ production are less inhibitory compared to control cells (25-40% decrease in inhibition depending on the content of DCreg in culture, P<0.05, ANOVA), but still retain suppressor activity. (B) - Both B6 and I/St cell-free supernatants possessed inhibitory activity regarding T cell proliferation, but in I/St this activity is weaker (P<0.01, ANOVA). (C) - NO production: similar nitrite levels in B6 and I/St co-cultures with lung stroma from naïve mice and opposite shifts in the presence of infected stroma – slight decrease in I/St, but 2-fold increase in B6 cultures, resulting in 3-fold (*P*<0.01, ANOVA) differences. (D) - The content of IL-10 is significantly (P=0.04, Student’s *t*-test) higher in B6 compared to I/St supernatants developed on infected stroma. Mean triplicate CFU counts ± SD from 3 independent experiments are displayed.

In earlier studies it was firmly established that DCreg release their suppressive potential by secreting a number of soluble factors capable to inhibit T cell responses, e.g., PGE_2,_ IL-10 and NO ( [15,17]; reviewed in [19]). To evaluate possible inhibitory activity of PGE_2_ in our experiments, we developed DCreg on lung stroma in the presence (10µg/ml) or absence of the prostaglandin synthesis inhibitor indometacin and compared their capacity to inhibit T cell proliferation. As shown in Figure 4A, DCreg with blocked PGE_2_ production were less inhibitory compared to control cells (25-40% decrease of inhibition depending on the content of DCreg in culture, P<0.05, ANOVA), but still retained notable suppressor activity.

To identify other soluble inhibitory factors in our system and possible influence of infection on their production, we first assessed whether supernatants from co-cultures in which DCreg were developed possessed suppressive activity. Cell-free supernatants from co-cultures of DCreg precursors with stromal cells obtained from naïve and infected animals were collected at day 7 and titrated in our standard T cell proliferation system. As shown in Figure 4B, both B6 and I/St supernatants possessed inhibitory activity regarding T cell proliferation. However, in I/St this activity was weaker (P<0.01, ANOVA) compared to B6 mice. Moreover, when stroma from infected mice was used to instruct DCreg differentiation, inhibitory capacity of co-culture supernatants followed the pattern characteristic for DCreg themselves: diminished in I/St but did not change in B6 preparations.

In addition to PGE_2_, another low molecular weight inhibitor of T cell activation and proliferation is nitric oxide [26]. To find out whether DCreg cells from the two mouse strains differ in NO production, we evaluated its level in co-culture supernatants. As shown in Figure 4C, there was little difference in nitrite levels when DC were co-cultured with lung stroma from naïve mice. However, DC differentiation in the presence of infected stroma shifted the NO level in opposite directions: it slightly decreased in I/St, but increased 2-fold in B6 cultures, suggesting that interstrain differences in capacity to inhibit T cell responses may be due, likely in part, to changes in NO production following infection.

The key cytokine which is actively produced by DCreg and inhibit type 1 T cell activation is IL-10 [27]. To assess its possible role in our system, we measured the IL-10 concentrations in supernatants and found that the content of IL-10 was significantly (P=0.04, Student’s *t*-test) higher in B6 cultures developed on infected stroma (Figure 4D), which is in line with other results described in this section. Thus, following infection lung stroma of TB-resistant B6 mice retained its ability to instruct DCreg, contrary to what was seen in TB-susceptible I/St mice. Suppressive effect provided by DCreg was released via a few soluble mediators inhibiting T cell proliferation.

Control experiments with supernatants obtained from cultures containing only stromal cells demonstrated no suppressor activity (Figure S3A), and these supernatants contained only marginal levels of PGE_2_, NO and IL-10. However, when stromal cells were directly added to T cell proliferative system they displayed a profound suppressor activity (Figure S3B) demonstrating the multitude of mechanisms providing T cell response inhibition in the lung.

### B6 and I/St mice differ by the content and dynamics of regulatory T cells in lymph nodes

There is ample evidence that regulatory DC induce generation of CD4^+^CD25^+^Foxp3^+^ regulatory T cells ( [15,28,29]; reviewed in [3]), and that chronic infections strongly influence this regulatory pathway (reviewed in [4,27]). With this regard, it was interesting to find out whether TB-susceptible I/St differ from TB-resistant B6 mice by the content of Treg cells during chronic TB infection. To address this issue, we estimated the content of CD4^+^CD25^+^Foxp3^+^ cells along slowly progressing TB infection (~100 CFU administered as aerosol) in mediastinal lymph nodes which drain the lungs.

**Figure 5:**
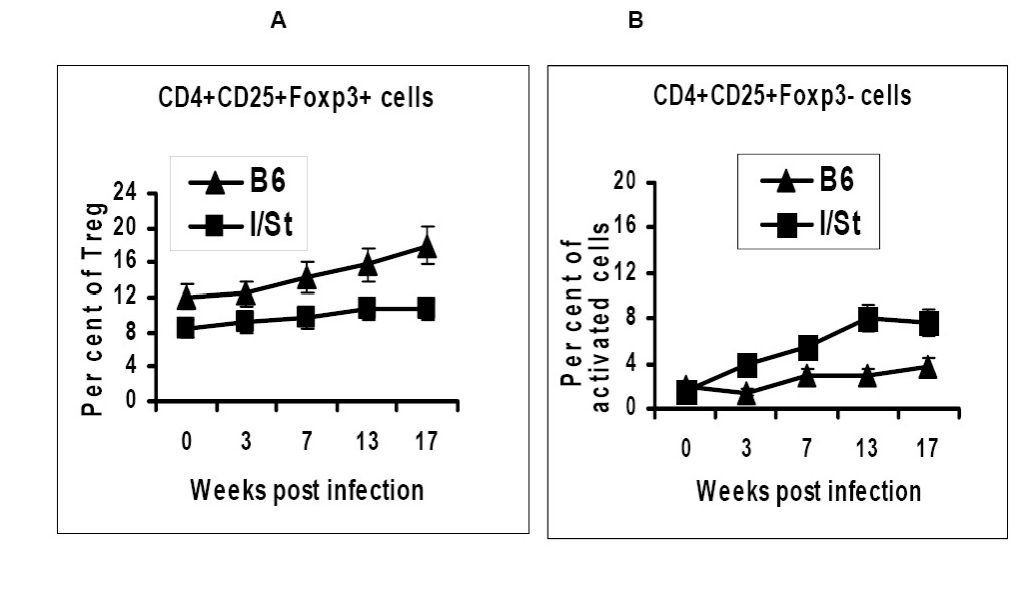
B6 and I/St mice differ in numbers (*P*< 0.05-0.01 at different time points, Student’s *t*-test) of regulatory (A) and activated (B) CD4^+^ T cells in mediastinal, lung-draining lymph nodes throughout the course of infection.

As shown in Figure 5, the content of Treg cells was significantly (*P*<0.05, Student’s *t*-test) higher in lymph nodes of B6 compared to I/St mice even before challenge (week 0, Figure 5A). As infection progressed, Treg population remained stable in I/St, but slowly increased in B6 animals, reaching approximately 2-fold difference (*P*<0.01, Student’s *t*-test) by week 17 of infection. Contrary, the population of activated CD4^+^CD25^+^Foxp3^-^ grew faster and reached higher numbers in I/St compared to B6 mice (Figure 5B).

## Discussion

As emphasized in a recently published review [19], there are large gaps in our knowledge regarding the identity of regulatory DC. It is not clear whether regulatory DC are terminally differentiated cells or represent a transient functional state of a more general DC population triggered by the contact with stroma in an environment of organs. In particular, more should be learned about modulation of stromal cell function by pathogens and inflammatory reactions to consider therapeutic strategies aimed at DC manipulation.

So far, shifts in instructive activity of stromal cells regarding immature DCreg development during host-parasite interactions were demonstrated only for leishmania [17] and helminth [30] invasions. We add intracellular mycobacterial pathogens to this list by demonstrating that TB infection differentially affects instructive capacity of lung stroma in genetically TB-susceptible and resistant mice, and that the developing DCreg directly down-regulate the response of mycobacteria-specific CD4^+^ T lymphocytes. Our findings demonstrate that both major cell populations functioning as down-regulators of superfluous T cell immune responses, DCreg (Figure 3) and Treg (Figure 5), were more readily developed and more efficiently maintained in mice displaying less severe lung inflammation and pathology in the course of TB infection.

These results are in good agreement with those recently published by Leepiyasakulchai et al. [20] who demonstrated similar correlations between the numbers of regulatory dendritic and T cells in the lung, genetic susceptibility to TB challenge and degree of lung pathology. Importantly, their observations concerned a different population of regulatory DC, namely, anti-inflammatory CD103^+^CD11b^-^CD11c^+^ αE-DC [21], whereas in our study we characterized immature CD11b^+^CD103^-^ DCreg educated on lung stroma. Yet another example of pneumonic phagocyte’s involvement in immune response inhibition which depends upon mycobacterial infection is an enhanced production of TGF-β and PGE_2_ by pleural macrophages following engulfment of BCG-infected apoptotic neutrophils [31]. Given that the macrophage-rich lung stroma itself is able to directly inhibit T-cell immune responses to several infectious and polyclonal stimuli ( [35], and Figure S3), we may conclude that a redundancy in the T cell responses suppression system is an essential feature of the lung tissue functioning. This is not surprising, since maintenance of the balance between cellular immune responses-mediated protection and pathology is critical for successful regulation of overwhelming inflammation affecting mucosal organs during chronic pathologic conditions, such as TB and IBD [32,33].

Whereas cell populations participating in inhibition of immune responses and inflammation in the lung are impressively diverse, the number of soluble mediators providing suppression which are produced by these cells is small. Production of anti-inflammatory cytokine IL-10 is a feature conserved among virtually all systems in which activity of immune response inhibitory cells, such as different regulatory DC and Foxp3^+^ Treg, has been studied. So far, the reliable list of inhibitory mediators is limited to IL-10, TGF-β, NO and PGE_2_ (3,19). Our results are in agreement with previous findings: inhibitory activity of supernatants obtained from DCreg educated on lung stroma partly depended upon the presence of PGE_2_, and the differences in regulatory activity of DCreg obtained from naïve and TB-infected mice of susceptible and resistant strains correlated with the differences in IL-10 and NO production (Figure 4). It was not possible to compare the levels of TGF-β in supernatants in our system: expansion of DCreg in co-cultures with lung stroma is ineffective in the absence of FCS in culture medium, ELISA results are strongly altered due to the presence of FCS, and qrt-PCR says little about real secretion of the active form of TGF-β.

In summary, our data demonstrate that the lung stroma educates DC progenitors in such a way that they develop in DCreg able to inhibit mycobacteria-specific T cell proliferation. This ability is apparently important for limiting inflammation caused by *M. tuberculosis*-triggered disease, which is underlined by the consequence of infection in genetically different mouse strains: during the period of observation instructive capacity was retained by more resistant mice but gradually decreased in TB-susceptible animals.

## Materials and Methods

*Mice* of inbred strains I/StSnEgYCit (I/St) and C57BL/6JCit (B6) were bred and maintained under conventional, non-SPF conditions at the Animal Facilities of the Central Institute for Tuberculosis (CIT, Moscow, Russia) in accordance with guidelines from the Russian Ministry of Health # 755, and under the NIH Office of Laboratory Animal Welfare (OLAW) Assurance # A5502-11. Water and food were provided *ad libitum*. Female mice of 8-12 wk of age in the beginning of experiments were used. All experimental procedures were approved by the CIT animal care committee (IACUC protocols 2, 7, 8, 11, 13 approved on March 6, 2011).

### Infection

To evaluate severity of the disease and to create the source of infected lung stroma, mice were infected with 1-2 x 10^2^ CFU of standard virulent *M. tuberculosis* strain H37RV (sub-strain Pasteur) using an Inhalation Exposure System (Glas-Col, Terre Haute, IN) exactly as described earlier [9]. To assess CFU counts, lungs from individual mice were homogenized in 2.0 ml of sterile saline, and 10-fold serial dilutions of 0.2 ml samples were plated on Dubos agar (Difco) and incubated at 37^0^C for 20-22 days. To examine pathology of the lung tissue, mice were euthanized by the thiopental (Biochemie GmbH, Vienna, Austria) overdose. Lung tissue (the middle right lobe) was frozen in the regimen of -60^0^C to -20^0^C temperature gradient in the electronic Cryotome® (Thermo, Shandon, UK), and 6-8µm-thick sections were made across the widest area of the lobe, fixed with acetone and stained with hematoxylin-eosin.

### Lung stromal cell preparations

Lung stroma from 4 donor mice of each strain was obtained in each experiment. Naïve or infected B6 and I/St mice were euthanized by injection of the thiopental overdose, and lung cell suspensions were prepared using the methods described earlier [34]. Briefly, blood vessels were washed out and repeated broncho-alveolar lavage was performed using 0.02% EDTA-PBS with antibiotics. Lung tissue was sliced into 1-2 mm^3^ pieces and incubated at 37^0^C for 90 min in RPMI-1640 containing 5% FCS, antibiotics, 10 mM HEPES (all components – HyClone, Carlington, the Netherlands), 200 U/ml collagenase and 50 U/ml DNase-I (Sigma). Single cell suspensions were obtained by vigorous pipetting. Lung cells were washed twice in HBSS containing 2% FCS and antibiotics and re-suspended in RPMI-1640 medium supplemented with 10% FCS, 10 mM HEPES, 2 mM L-glutamine, 1% non-essential amino acids, 1 mM pyruvate, 5x10^-5^ 2-mercaptoethanol and antibiotics (all components - HyClone). 20-30 x 10^6^ cells were incubated in 10 ml of medium 1 for 2 h on 90 mm Petri dishes (Costar-Corning, Badhoevedorp, the Netherlands) at 37^0^C. Non-adherent cells were removed by triple washing with warm HBSS containing 2% FCS. Adherent cells were detached from plastic by incubating monolayers in 0.02% EDTA-PBS solution for 30 min at room temperature, and after triple washing re-suspended in supplemented RPMI-1640 for further culturing.

### Purification and culture of progenitor cells

Bone marrow cells were obtained by aspirating femur bones from 12–16 mice of each strain in each experiment. BMLin^-^ cells were isolated by using a cell lineage depletion kit (Milteneiy Biotech, Gladbach, Germany) according to manufacturer’s protocol. Briefly, bone marrow cell were labeled with biotinylated antibodies to CD11b, CD5, CD45R, Gr-1 (Ly6C + Ly6G), 7-4 and TER-119 surface markers for 10 min in PBS containing 0.5% BSA and 2mM EDTA (Sigma, MS). The cells were incubated with anti-biotin-conjugated immune magnetic beads at 4^0^C for 15 min in PBS containing 0.5% BSA and 2mM EDTA. After washing, lineage-positive cells were removed on MidiMACS separation columns, and lin^-^ cells were collected. The yield of lin^-^ cells was ~2% of the total bone marrow cell numbers. 50 x 10^3^ BMLin^-^ cells were cultured together with 10 x 10^3^ lung stromal cells in 96-well plates for 7 days in supplemented RPMI-1640, and the number of non-adherent viable cells approximately doubled by the end of culture.

*DC surface phenotypes* were assessed using the following labeled antibodies from BD-Pharmingen, San Jose, CA: anti-CD11b-PE (clone ICRF44), anti-CD11c-APC (clone B-ly6), anti-CD80-FITC (clone B7-1), anti-CD103-FITC (clone M290), anti-Gr-1-FITC (clone RB6-8C5), anti-IA^p^-FITC (clone 7-16.17, reacts with IA^j^), anti-IA^b^-PE (clone AMS-32.1).

### Stimulation/rest protocol for developing T cell lines

B6 and I/St mice were immunized into rear foot pads with 10µg/pad of *M. tuberculosis* H37Rv water-soluble antigenic fraction obtained by ultrasound disintegration and mixed with incomplete Freund’s adjuvant. Cells were cultured as described previously [35]. Briefly, 2 x 10^6^ immune cells isolated from popliteal lymph nodes were cultured in 1 ml RPMI‑1640 containing 10% FCS, 10 mM HEPES, 4 mM L-glutamine, 5 x 10^-5^ M 2-ME, vitamins, piruvate, non-essential amino acids and antibiotics (HyClone) in 24-well plates (Costar) for 14–16 days in the presence of 10µg/ml mycobacterial sonicate. Immune cells (>95% viability by trypan blue exclusion) were isolated by centrifugation at 2500 g for 20 min at 23^0^C, on a 1.088 g/ml Lympholyte M gradient (Cedarlane Labs, Ontario, Canada), washed twice and counted. The next stimulation cycle was accomplished by co-culturing 2 x 10^5^ isolated cells with 1.0 x 10^6^ mitomycin C-treated syngenic splenic APC and sonicate for another 14-16 days. These cycles were repeated 4-5 times until the cells (99% CD4^+^ by flow cytometry) started to grow as a stable cell line. Cells were frozen and stored at -150^0^C until used in proliferation assays.

### Proliferation assay

10^4^ T line cells were co-cultured with 2 x 10^5^ mitomycin C-treated splenic APC in a well of 96-well flat-bottom plate (Costar), at 37^o^C, 5% CO_2_, in supplemented RPMI-1640 containing 5% FCS. Cells were stimulated with 10 µg/ml of H37Rv sonicate. All cultures were performed in triplicates and non-stimulated wells served as controls. DCreg cells or their cultural supernatants were added at indicated concentrations from the beginning of assay. Cultures were pulsed with 0.5 µCi of [^3^H]-thymidine for the last 18 h of 40 h incubation. The label uptake was measured in a liquid scintillation counter (Wallac, Turku, Finland) after harvesting the well’s contents onto fiber glass filters using a semi-automatic cell harvester (Scatron, Oslo, Norway).

### Soluble mediators

The content of IL-10 and NO was measured in supernatants at day 7 of co-culturing DCreg progenitors with lung stroma. OptEIA mouse IL-10 Set from BD-PharMingen (sensitivity -63 pg/ml) was used to measure IL-10 using ELISA format. The concentration of nitrite, a stable metabolite of NO, was determined using Griess reaction [36], with measuring absorbance at 550 nm in a micro-ELISA reader (Sigma), using a 620-nm reference filter.

## Supporting Information

Figure S1 Lung stroma of B6 and I/St mice has similar cellular composition and contains ~7% ER-TR7+ fibroblasts, ~15% CD31^+^ endothelial cells and >50% resident CD68^+^ macrophages.
(TIF)

Figure S2 Precursors of DCreg cells isolated from bone marrow of B6 and I/St mice using lineage depletion kit comprise ~60% of c-kit^+^ cells.
(TIF)

Figure S3 Stromal lung cells from naïve and infected B6 and I/St mice (A), but not their culture supernatants (B) inhibit antigen-specific proliferation of T cell lines.
(TIF)
