# Supplementary figures and images for: Capacity of Lung Stroma to Educate Dendritic Cells Inhibiting Mycobacteria-Specific T-Cell Response Depends upon Genetic Susceptibility to Tuberculosis
Source: PLoS One. 2013 Aug 15;8(8):e72773. doi: 10.1371/journal.pone.0072773 (PMC3744498; doi:10.1371/journal.pone.0072773)

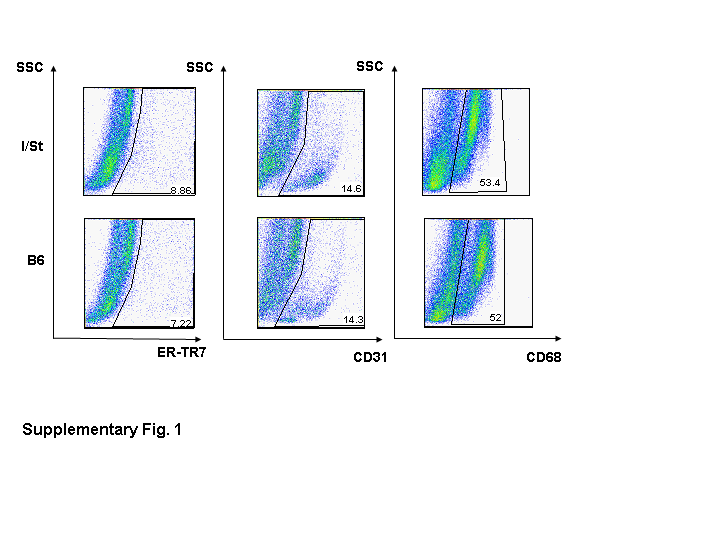

Supplement: Figure S1 — Lung stroma of B6 and I/St mice has similar cellular composition and contains ~7% ER-TR7+ fibroblasts, ~15% CD31+ endothelial cells and >50% resident CD68+ macrophages. (TIF) [file pone.0072773.s001.tif]

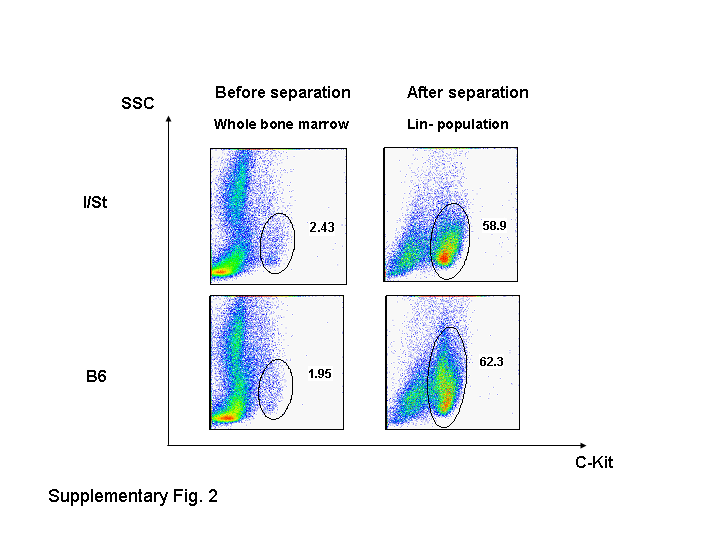

Supplement: Figure S2 — Precursors of DCreg cells isolated from bone marrow of B6 and I/St mice using lineage depletion kit comprise ~60% of c-kit+ cells. (TIF) [file pone.0072773.s002.tif]
